# Photocatalytic Activity of Nanotubular TiO_2_ Films Obtained by Anodic Oxidation: A Comparison in Gas and Liquid Phase

**DOI:** 10.3390/ma11040488

**Published:** 2018-03-24

**Authors:** Beatriz Eugenia Sanabria Arenas, Alberto Strini, Luca Schiavi, Andrea Li Bassi, Valeria Russo, Barbara Del Curto, Maria Vittoria Diamanti, MariaPia Pedeferri

**Affiliations:** 1 Department of Chemistry, Materials and Chemical Engineering “G. Natta” Politecnico di Milano, Via Mancinelli 7, Milan 20131, Italy; beatrizeugenia.sanabria@polimi.it (B.E.S.); barbara.delcurto@polimi.it (B.D.C.); mariapia.pedeferri@polimi.it (M.P.); 2 ITC-CNR, Construction Technologies Institute, Viale Lombardia 49, San Giuliano Milanese, Milan 20098, Italy; alberto.strini@itc.cnr.it (A.S.); luca.schiavi@itc.cnr.it (L.S.); 3 Department of Energy, Politecnico di Milano, Via Ponzio 34/3, Milan 20133, Italy; andrea.libassi@polimi.it (A.L.B.); valeria.russo@polimi.it (V.R.)

**Keywords:** nanostructured materials, titanium dioxide, anodizing, photocatalysis, toluene, rhodamine B

## Abstract

The availability of immobilized nanostructured photocatalysts is of great importance in the purification of both polluted air and liquids (e.g., industrial wastewaters). Metal-supported titanium dioxide films with nanotubular morphology and good photocatalytic efficiency in both environments can be produced by anodic oxidation, which avoids release of nanoscale materials in the environment. Here we evaluate the effect of different anodizing procedures on the photocatalytic activity of TiO_2_ nanostructures in gas and liquid phases, in order to identify the most efficient and robust technique for the production of TiO_2_ layers with different morphologies and high photocatalytic activity in both phases. Rhodamine B and toluene were used as model pollutants in the two media, respectively. It was found that the role of the anodizing electrolyte is particularly crucial, as it provides substantial differences in the oxide specific surface area: nanotubular structures show remarkably different activities, especially in gas phase degradation reactions, and within nanotubular structures, those produced by organic electrolytes lead to better photocatalytic activity in both conditions tested.

## 1. Introduction

Heterogeneous photocatalysis using semiconductors is an inexpensive and environmentally friendly treatment technology for degrading organic and inorganic pollutants in gas and liquid phase, which presents several advantages, such as minimal equipment and reuse of catalyst [1,2]. The number of photocatalytic studies reporting liquid phase reactions is far higher than those related to gas phase; however, this tendency has started to change in recent years, due to different applications of this technology in indoor environments like office buildings, factories, aircraft and spacecraft [3]. In such environments, the biggest concerns are related not only to pollution coming from outdoor activities—such as NO_x_ produced by vehicles and heaters combustion processes—but also from inside: paints, varnishes, detergents and furniture are all sources of volatile organic compounds, such as benzene, toluene and formaldehyde, which pose health risks to the inhabitants, as stated by WHO—World Health Organization guidelines for indoor quality.

On the one hand, a strong correlation between chemical nature of pollutant, catalyst surface characteristics, concentration of water vapour and obtained photoactivity has been reported in gas-solid photoreactions [4]. Einaga et al. [5] studied the degradation of toluene at different concentrations and water vapour content and reported a dependence between the initial concentration of toluene and the type of intermediates formed, as well as the necessity of water vapour in the system for complete mineralization of the intermediates to CO_2_ and CO. Nonetheless, one of the main challenges in this type of photocatalysis is to avoid the deactivation of the catalyst due to the accumulation of decomposition products at the surface of the substrate [6]. On the other hand, in liquid phase the number of variables is greater than in gas phase, and therefore the complexity. Surface composition, surface area, preparation procedures, concentration of catalyst, pH, concentration and chemical structure of the pollutant, partial pressure of oxygen and diffusion rate, are some of the parameters that can influence the photocatalysis of solid-liquid reactions [7]. Yet, photocatalysis has proved to be an effective technique for both water and air purification purposes, and several materials are available for the task, with different characteristics, depending on the specific application: from TiO_2_ to ZnO, from WO_3_, to SnO_2_. These materials are available in form of nanoparticles, nanowires or nanotubes, and modifications of chemical compositions can enhance the activity of these materials making them suitable also for visible light applications [8,9].

Most of the studies about photocatalytic degradation with TiO_2_ in both liquid and gas phase have been performed using commercial available nanopowders like AEROXIDE^®^ TiO_2_ P25, previously known as Degussa P25, which is either chosen as model photocatalyst, or used as comparison due to the large amount of data available on this material [10,11,12,13,14,15]. The dispersion of nanoparticles in liquid phase makes it necessary to add one or more recovery steps after photocatalysis, increasing the process costs and complexity, especially at industrial scale [16,17,18]. Other problems regarding the use of nanopowders are agglomeration in aqueous suspensions, which decreases photocatalytic activity, and light scattering caused by the same nanoparticles, which limits light absorption in large reactors [2]. 

Immobilized nanoparticles have been proposed as solution to this problem. This can be done over different materials [3] but glass has been by far the most used because it allows the penetration of light. However, the impossibility to use organic matrices such as common paints due to their photocatalytic destruction [1], lower photocatalytic activity due to decrease in surface area and light absorption [19], as well as limited mass diffusion of reactants within an immobilizing medium—be it organic or inorganic—are some of the disadvantages found by using this methodology, requiring the use of more resistant binders, such as perfluorinated ones [20,21]. 

Photocatalytic nanoparticles can also be immobilized onto inorganic substrates by high-temperature sintering, as example after deposition from a slurry [18] or by a suitable film-making process such as screen-printing [22]. However, TiO_2_ sintering is strongly limited by the anatase phase thermal instability, as it converts to the far less active rutile above 500 °C, preventing to reach the higher temperatures needed to ensure an optimal film strength. Improvements can be obtained by doping the anatase with suitable metal cations such as Zr^4+^ [23] that act as phase stabilizers. This can be done during the nanoparticle synthesis or also with undoped anatase during the sintering itself, but the process temperature is anyway limited to ~800 °C in order to avoid a significant degradation of the photocatalytic activity [24].

Anodic oxidation is a powerful technique that allows the production of various types of oxides on the surface of titanium [25], including highly ordered nanotubular oxides with comparable or higher photocatalytic activity than nanoparticles [26,27], and strong adherence to a metallic substrate [19]. Such structures are produced by anodizing the metal in solutions containing fluorides (or, more broadly, halogens), which dissolve the oxide locally while it is growing, allowing the formation of self-organized nanopores or nanotubes. This technique allows to tune very important parameters such as pore diameter, tube length, oxide thickness, wall smoothness, and porosity which affects physical, electronic, and chemical properties that finally have repercussions in the photocatalytic efficiency [28,29,30,31,32,33,34]. After nanotubes anodizing it is mandatory to anneal the sample at temperatures between 300 and 500 °C to induce oxide crystallization to anatase phase, which is acknowledged to be more efficient than rutile, owing to its higher charge carrier mobility [16]. On the other hand, anodizing at high voltages in sulfuric acid or other electrolytes allows direct crystallization of the oxide during its growth, on account of the onset of anodic spark deposition conditions: the higher the voltage, the higher anatase and rutile contents in the oxide, accompanied by a sub-micrometric porosity [35]. 

The lack of reproducibility, however, is still the main problem for this new technology to find a widespread use in industrial applications. In order to overcome these difficulties, clear correlations between anodizing parameters, material characteristics and obtained photoactivity should be thoroughly investigated, According to Zhuiykov [36] the morphology, crystal structure, surface stoichiometry, catalytic activity and chemical composition can be affected by the process used to produce the nanostructures and it explains why it is so difficult to reproduce the results obtained by other researchers. In other words, a particular fabrication process can affect the pathway that the molecules follow during degradation. 

For these reasons, this work proposes a comparison among several different anodizing treatments featuring different process parameters, which lead to different oxide morphology and crystallinity. All of them were tested in the photocatalytic degradation of liquid pollutants, in particular the organic dye rhodamine B (RhB), and in gas phase photocatalytic degradation of toluene. This is meant to provide a reliable comparison among different anodizing techniques and procedures, as all tests are performed in a homogeneous way, thus solving the issue of comparing results provided by different research studies. Three categories of anodic oxides were chosen: anodic spark deposition (ASD) oxides, nanotubes produced in aqueous solution and nanotubes obtained in organic solution. The final aim of this work is to present a robust method to obtain immobilized TiO_2_ nanostructures by anodic oxidation, and to allow for the most suitable anodizing choice as a function of the desired application, be it gas or liquid phase, taking into account method robustness and ease of reproduction.

## 2. Materials and Methods

### 2.1. Chemicals and TiO_2_ Nanostructures Synthesis

All tests were performed on commercial purity titanium sheets (grade 2 ASTM), employing NaF (Fluka), ethylene glycol (Fluka) and H_2_SO_4_ (Merck) for the electrolytes, and studying the photocatalytic degradation of Rhodamine B (Sigma) and toluene (diluted standard cylinder in N_2_, SIAD, Italy) in presence of the anodized titanium. All the reagents used were analytical grade.

Anodic oxidation was made in three different conditions, selected on the basis of previous preliminary evaluations [37,38]:

- In 0.5 M H_2_SO_4_ by applying 150 V for 2 min (label: ASD, anodic spark deposition);
- In NaF + Na_2_SO_4_ at 20 V, maintained constant for 6 h, followed by annealing at 400 °C for 2 h (label: A-NT, aqueous nanotubes);
- In NH_4_F + ethylene glycol (EG) at 45 V maintained constant for 30 min, followed by annealing at 400 °C for 2 h (label: O-NT, organic nanotubes).

The effect of electrolyte has been taken into particular consideration, as it was found to have an important impact on TiO_2_ nanostructuring and photocatalytic performance [38]. In order to induce the crystallization of nanotubular oxides, which are natively amorphous, annealing was performed at a temperature of 400 °C for 2 h. On the other hand, anodic oxides grown in ASD conditions already show a crystalline nature, therefore annealing was not performed, which allowed identify this preparation procedure as the least onerous from the point of view of both time and energy requirement.

### 2.2. Characterization

Scanning electron microscopy (SEM) was performed on a Carl Zeiss AG-EVO^®^ Series 50 (Carl Zeiss AG, Oberkochen, Germany). Glow discharge optical emission spectroscopy (GDOES) was also performed on ASD samples to estimate oxide thickness, using a Jobin-Yvon RF-GDOES profilometer(Horiba, Fukuoka, Japan) Raman spectra were acquired using a InVia micro Raman spectrophotometer(Renishaw, Gloucestershire, UK) with 514.5 nm laser excitation wavelength and power on sample of about 1 mW. 

### 2.3. RhB Photocatalytic Degradation

Tests were performed as reported in a previous work, by immersing a 6.0 ± 0.5 cm^2^ sample in a beaker containing 25 mL of 10^−5^ M RhB [38] and irradiating samples for 6 h with a combined incandescent/Hg vapour lamp (Osram Vitalux, UV-A intensity of 3000 µW/cm^2^, Munich, Germany). Dye discoloration was taken as reference of its degradation at its peak light absorption (550 nm), and was followed on a Spectronic 200E spectrophotometer. (Thermo Scientific, courtaboeuf, France) To ensure the dye molecule actually underwent breakup of the conjugated structure and not only deethylation [39], the whole wavelength range of deethylated products absorption (500–550 nm) was monitored: no deethylation was observed in none of the cases under investigation, therefore only data related to the 550 nm peak are presented.

RhB degradation was evaluated by measuring the dye concentration (*C*) over time using optical absorbance (*Abs*) and the Beer Lambert equation (Equation (1)):

*Abs* = *εlC* (1)

where *l* and *ε* are optical constants related to the measurement equipment (length of optical path) and reactant (optical absorption constant), respectively. Adsorption measurements performed in dark storage, monitoring in time the solution concentration after immersing the samples for a duration of 3 h, showed negligible adsorption of the dye onto the TiO_2_ surface, as attested by absorbance values decreasing of approximately 1%. In photocatalytic tests, irradiation started after 20 min from sample immersion. Then data were processed considering a pseudo first order reaction kinetics, as typical of dye degradation reactions [38]. Data were then expressed as reaction rate constant per unit area (*k_app_*) obtained from the following relationship (Equation (2)):

(2) $$\frac{\ln\left(\frac{C}{C_{0}}\right)}{A}=-k_{app}t$$

where *C_0_* is initial RhB concentration, *A* is the sample surface area and *t* is irradiation time, in order to compare photocatalytic activity values normalized by each specimen area.

### 2.4. Toluene Photocatalytic Degradation

Toluene degradation (0.75 µmol m^−3^) in air (25 °C and 50% RH) was assessed in a continuous stirred tank photoreactor (CSTR) operating at predetermined pollutant concentration. The desired toluene concentration inside the photochemical reactor was reached for each measurement by a two-step successive approximation process under UV irradiation, as detailed in a previous work [40]. This approach allows performing all the measurements at the same nominal operating concentration without any dependence on the actual sample activity. The computer-controlled experimental system includes an artificial air generator, an irradiation chamber that provides for photoreactor thermal and irradiance control and a GC/PID analyzer. The artificial air generator comprises four digital mass flow controllers with ± 0.25% nominal repeatability (model 5850, Brooks, Seattle, WA, USA) that deliver the required amount of pure nitrogen and oxygen from GC quality distribution lines (SIAD, Bergamo, Italy) and toluene (prediluted in nitrogen) from a standard cylinder (SIAD). Humidification is provided by a dedicated nitrogen line bubbled in bidistilled water. The reactor was operated with 1800 rpm mixing fan speed for all photocatalytic activity measurements. The irradiation chamber was equipped with two or six fluorescent lamps (PL-S/BLB, Philips, Amsterdam, The Netherlands) as UV-A source resulting respectively in 160 or 525 µW cm^−2^ irradiance at the sample surface (340–400 nm range), selected accordingly to the experimental needs. Toluene analyses were performed by an automated GC/PID system provided with thermal desorption sampler/concentrator (GC955, Synspec, NL, Groningen, Netherlands). All gas samples were taken downstream the photoreactor after equilibration (i.e., in steady-state conditions); the reactor inlet concentration was measured at the reactor outlet with irradiation turned off. The photocatalytic activity was expressed as toluene area-specific degradation rate in air, as expressed in Equation (3) (*r_a_*, in mol m^−2^ s^−1^):

(3) $$r_{a}=\left(\frac{Q}{A}\right)\left(C_{0}-C\right)$$

where *Q* is the volumetric flow rate (m^3^ s^−1^); *A* is the sample area (m^2^); *C_0_* is the inlet concentration of toluene (mol m^−3^) and *C* its concentration in the reactor (mol m^−3^).

The degradation rate measurement repeatability was calculated as error propagation in Equation (3) assuming ± 2% error in the concentration measurement *C*_0_ and *C*, ± 1% error in the supply air volumetric flow *Q* and ± 3% error in the sample surface A [22], considering at least three tests per anodizing type.

## 3. Results

### 3.1. Anodic Oxides Characterization

**Figure 1:**
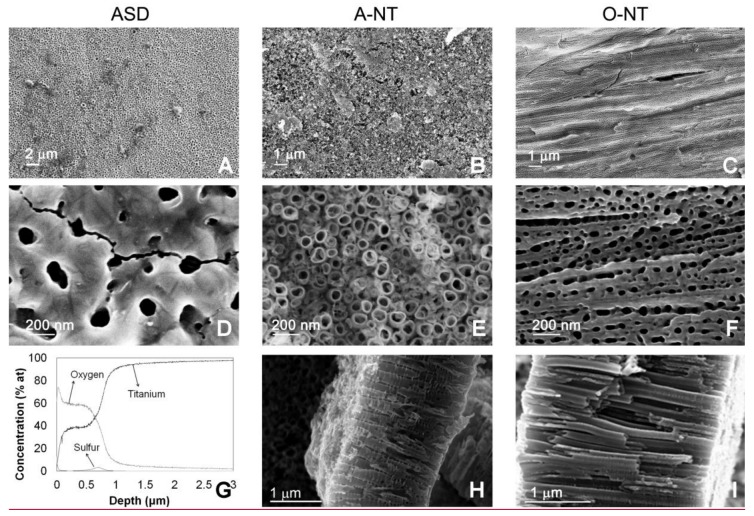
SEM micrographs of morphologies deriving from ASD (**left**), anodizing in aqueous solution with 4.5 h of anodizing (nanotubes, **center**) and in organic solution with 0.5 h of anodizing (nanopores/nanotubes, **right**) at different magnifications. The lower line reports information to derive oxide thickness: GDOES analysis for ASD samples, cross sections for nanotubes.

SEM and Raman analyses were carried out to understand the anodic oxides morphology and crystal structure. Figure 1 (left) shows a microscopically homogeneous surface, characterized at higher magnification by a glassy appearance, big pores and cracks, characteristic of ASD samples [35,41]. Oxide thickness is approximately 0.7 μm, as attested by GDOES analysis. For A-NT with 6 h of anodizing time (Figure 1 center), vertical nanotubes stem from the substrate with about 100 nm of diameter. Non-uniform tube length is observed, as proved by the clear three-dimensional roughness observed in the higher magnification image. The cross-sectional views show that oxide nanotube thickness (tube length) is in the range of 1.5 μm, with thinner sections in some points, which is in the expected range of length for this kind of electrolyte, as already reported by other authors [42]. In organic solution (O-NT, Figure 1 right), from the top view image, no clear separation or detachment of single nanotubes is visible, apparently a nanoporous template rather than a nanotubular array was created, with non-uniform tube diameter ranging from tens to hundreds of nm. Yet, the cross section demonstrates that it actually consists of well-defined single tubes with smooth walls, where only the top part is continuous. Tube length is in the order of 3 μm and the thickness of the walls is about 35 nm (Table 1). For this reason, and for the sake of simplicity, these oxides will also be referred to as nanotubular. More details on the analysis of pore dimensions and distribution is reported in the discussion section.

**Figure 2:**
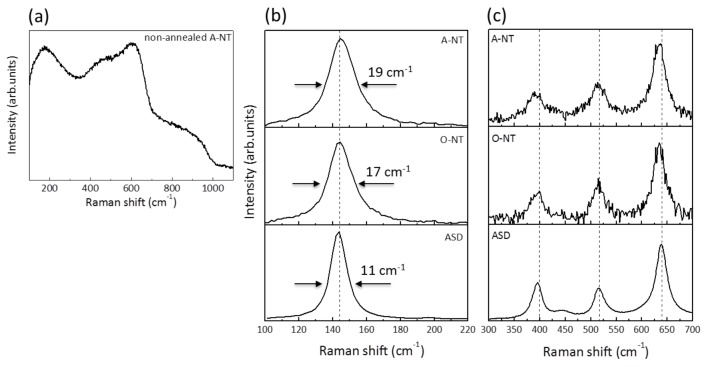
(**a**) Raman spectrum of non-annealed A-NT; (**b**,**c**) Raman spectra of the three samples in Table 1, as prepared ASD, A-NT and O-NT after annealing at 400 °C. Because of the very high intensity of the peak at 144 cm^−1^ with respect to all other peaks, spectra are separated into two panels with arbitrary intensity units. Vertical lines indicate reference peak positions for anatase crystal (144, 399, 517, 638 cm^−1^). The width of the peak at 144 cm^−1^ is extracted by fitting with a lorentzian curve.

Structural characterization of the three kinds of anodic oxides was performed by Raman spectroscopy (Figure 2). ASD samples were analyzed without further treatment, confirming the predominance of anatase phase with sharp peaks at the expected positions (144, 399, 517, 639 cm^−1^). Moreover, the high signal to noise ratio and the reduced width of the peaks attest a good degree of crystallinity of this anodic oxide. We underline also the presence of a further feature at about 430 cm^−1^, which is compatible with the presence of rutile traces. Moving to nanotubes, both as prepared A-NT and O-NT present a band-like spectrum in the 100–800 cm^−1^ range (Figure 2a) typical of amorphous oxides, while after annealing at 400 °C the anatase peaks appear. 

Comparing with ASD samples, the spectra of annealed nanotubes present lower signal to noise ratio and larger peaks, suggesting a lower degree of crystallinity. In particular, the width of the most intense peak (at 144 cm^−1^), as reported in Figure 2, varies from 11 cm^−1^ for ASD samples to 17–19 cm^−1^ for nanotubes, confirming the presence of a larger amount of disorder [43]. It has been documented that TiO_2_ crystalline structure has strong influence in the photocatalytic activity of the semiconductor, being anatase the most active phase [26]. On the other hand, the co-presence of anatase and a smaller fraction of rutile have also been proved beneficial for the overall TiO_2_ photoactivity [44,45,46]. X-ray diffraction measurements were also performed to double check the crystalline structures present in the oxides, which indeed was confirmed as provided by Raman characterization (data not shown).

### 3.2. Photocatalytic Activity

Aqueous nanotubes (A-NT) were produced in a previous work, and showed good activity and reproducibility in RhB degradation [38]; therefore, these nanostructures were first considered. Since these nanotubes required a long anodizing (6 h), which makes the process particularly time and energy consuming, an attempt was made in the direction of reducing this parameter. For this reason, nanotubes produced in a shorter time (30 min) in organic solution and porous oxides obtained in a very quick ASD process lasting only 2 min are here proposed as alternatives. ASD was also previously evaluated as anodizing technique and gave interesting results concerning morphology and crystal structure [47]. A high voltage giving the co-presence of anatase (main phase) and rutile (to a minor extent) was chosen, as in previous literature this combination of crystal phases was observed to give better results than the sole anatase phase [48]. On the other hand, nanotubes in organic solution were shown to allow higher tube length compared to aqueous ones in shorter times [16]. Yet, the electrolyte poor conductivity generates high ohmic dissipations and consequent electrolyte heating during anodizing, which can lead to issues in reaching high voltages: therefore, a low cell voltage was chosen, i.e., 45 V, in order to have a more repeatable process and allow the anodizing of larger areas, i.e., in the order of tens of square centimeters. This is considered fundamental in the optimization of a surface treatment if already aiming at real applications, where the production of photocatalytic reactors for water or air purification are addressed, where nominal areas of photoactive items should overcome the few mm^2^ that are often encountered in literature works.

**Figure 3:**
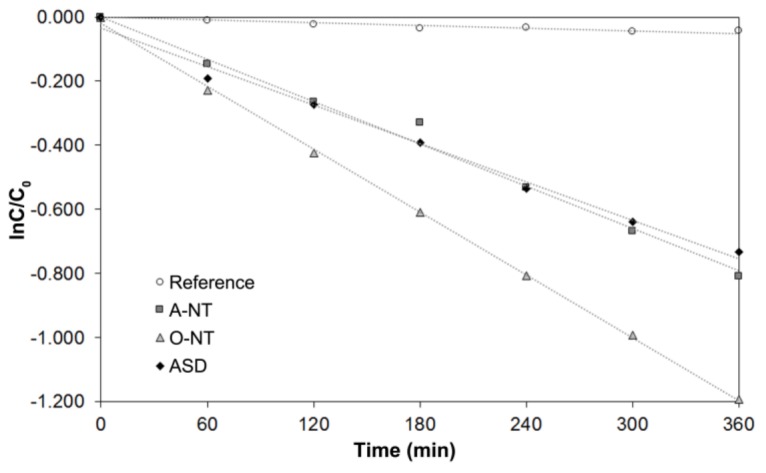
Kinetic plot for RhB degradation.

Figure 3 shows the degradation kinetics of RhB over 6 h irradiation, either in presence of anodized samples or without any photocatalyst (reference test). In the reference test there is no dye degradation, only a small decrease in absorbance due to photolysis. TiO_2_ samples all show a linear trend in ln(*C*/*C*_0_), confirming the hypothesized pseudo-first order kinetics of RhB degradation. Concerning a comparison among different oxides, ASD and aqueous nanotubes produce similar photocatalytic activity, while organic nanotubes clearly show an enhanced efficiency in RhB degradation.

Table 2 reports the photocatalytic degradation rates of both RhB and toluene. A first consideration on the photocatalysis tests reproducibility is here provided. Rhodamine b-based photocatalysis measurements were all conducted at least in triple, and uncertainties on the resulting photocatalytic activity are of the same order of magnitude, for all samples, i.e., 10% of the measured activity. The toluene measurement system had been previously characterized, and instrumentation-related measurement errors are in the order of 8% on the actual measurement, as reported in Table 2 data uncertainty. Moreover, concerning reproducibility, series of four nanotubular specimens prepared in the same batch were measured, giving a relative standard deviation of the measured gas-phase photocatalytic activity that was non separable from the analytical method repeatability (~8% in the given conditions). This indicates a less than 8% sample repeatability inside a single preparative batch. 

A huge influence of anodizing electrolyte, and therefore oxide morphology, was observed especially in gas phase, where ASD samples show negligible photocatalytic activity in spite of an efficiency comparable to aqueous nanotubes in liquid phase. To the best of our knowledge, this observation has not been reported before. The implications of this behavior can suggest that a significant part of the photodegradation process lies in a balance between the effects of morphology of the nanostructure, most noticeably specific surface area and its accessibility, and presence, type and amount of crystalline structures. The variation of sample relative reactivity is also evident comparing the A-NT and O-NT samples, with a remarkably higher difference in gas phase (about a factor ten), while in liquid phase only a 40% difference was observed between the two nanostructures.

**Figure 4:**
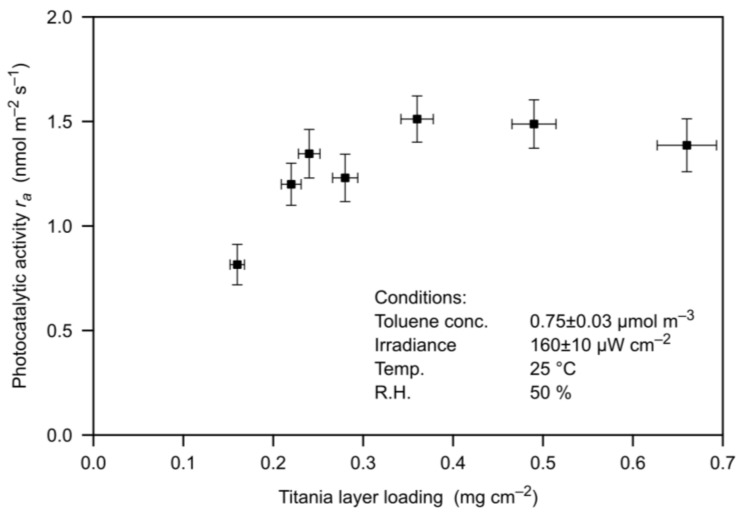
Photocatalytic toluene degradation activity of screen-printed titania samples made with Degussa Aeroxide P25 [22] in the experimental conditions indicated inside the figure.

In order to give a rough comparison with a well-know photocatalyst, Figure 4 reports the gas-phase toluene degradation activities of screen-printed samples made with Aeroxide-P25 (Degussa) characterized in a previously described research [22]. Tests were repeated in conditions coherent with the present work. Data indicate that the toluene catalytic activity demonstrated by the O-NT sample correlates to the activity of a P-25 screen-printed sample with about 0.2~0.3 mg cm^−2^ TiO_2_ layer loading. It is important to remark that such comparison is merely indicative, as it depends on the deposition technique conditions and consequent TiO_2_ layer parameters, such as degree of compaction and specific surface area available, which in turn influence the interaction with the effluent. Therefore this comparison holds only for the given measurement conditions.

## 4. Discussion

The presented results emphasize the variations in relative activity demonstrated by similar morphologies (i.e., nanotubes or pores) when utilized in gas or liquid phase photocatalytic processes. The different behaviors are possibly due to dissimilar physical constraints of the oxide matrices (e.g., reactant diffusivity), and they appear more conspicuous in the gas system, indicating a different exploitation of the catalyst in gas and liquid phase reactions. 

**Figure 5:**
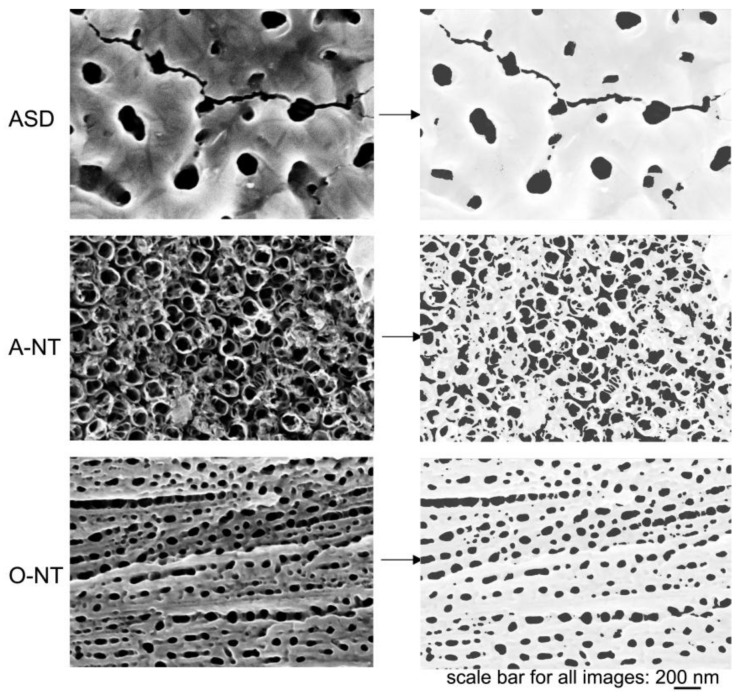
Topography of samples and highlight of open porosities.

**Figure 6:**
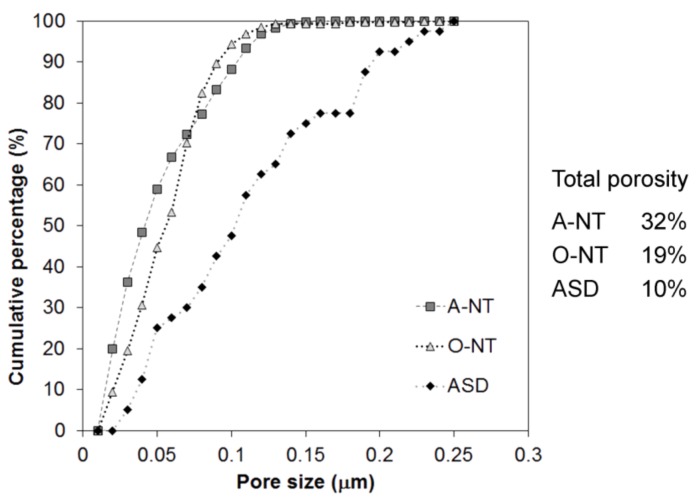
Analysis of porosities appearing on the three oxides surfaces.

It is worth comparing first the photocatalytic activity of the three materials in liquid phase. In this environment, differences are flattened: the overall dye degradation extent after 6 h of test ranges from 50% (ASD, A-NT) to 65% (O-NT), giving a difference of only 25% between the most and least effective oxides on the overall degradation percent, which increases to 40% if the reaction constants are taken into account. As previously pointed out, nanotubes produced in aqueous electrolyte and porous oxides obtained via anodic spark deposition present very similar activities, in spite of differences both in the crystallinity and in the surface area of the oxides. Yet, ASD samples exhibit higher crystallinity, which is beneficial to improve the separation and transport of photogenerated charge carriers, and also present a mixture of anatase and rutile phases which, as abovementioned, may show benefits compared to the pure anatase form [44,45,46]. On the other hand, nanotubes present a larger surface area, with double oxide thickness and larger amount of pores, in spite of a partial obstruction of some nanotubes. This is more clearly visible in Figure 5, where the surface topography has been re-addressed evidencing the darker areas—pore or tube openings—that contribute to the increase in surface area of these nanostructures with respect to the nominal one. The image was analyzed with Gwyddion [49] and pore size distribution was extracted and reported in Figure 6. Data analysis revealed a percentage of surface covered by pores triple in the A-NT sample, i.e., nanotubes produced in aqueous electrolyte, compared to ASD-obtained sample (32% vs. 10%, respectively), with an intermediate condition for the O-NT oxide grown in the organic electrolyte (19%). 

Therefore, the A-NT oxide can count on an overall double thickness and higher percent of pores with respect to the total oxide surface with respect to ASD: this increase in specific surface area is sufficient to counterbalance the lower oxide crystallinity.

On the other hand, O-NT samples also have a smaller percent of pore openings with respect to A-NT and same crystallinity (Figure 2), and should therefore present a lower photocatalytic activity, while on the opposite they showed the best performances in liquid phase photocatalysis. This was explained by considering the cross section of O-NTs, which is clearer from obstacles with smoother walls, as explained in the following.

**Figure 7:**
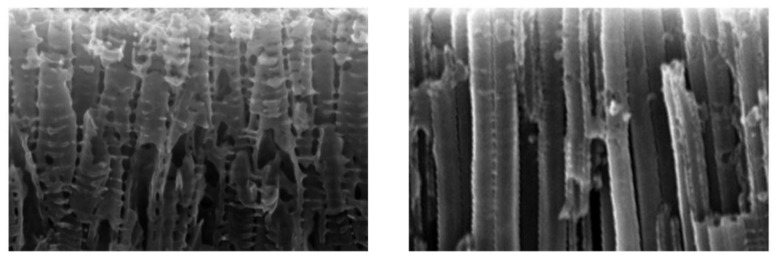
Schematic representation of nanotubes cross-section highlighting the tortuosity of A-NT.

In fact, Figure 7 reports a schematic representation of nanotubes cross-section highlighting the tortuosity of A-NT, which may be responsible of a shorter diffusion path of RhB molecules in aqueous nanotubes. It is evident that, in spite of a smaller overall number of pores, O-NT pores are more regular and freely accessible, with reference to the frequent partial pore occlusion visible in A-NT in Figure 4, which causes the appearance of a large percent of small porosity and the consequent reduction in average pore size. The difficulty for RhB molecules to diffuse through small pores and until the bottom of nanotubes would account for the higher photoactivity of nanotubes obtained in organic solution. Finally, ripples formation on the wall of nanotubes not only may obstruct available pores, but also act as charge carrier recombination points, therefore reducing activity: also this feature is more pronounced in nanotubes produced in aqueous electrolyte (A-NT) [50].

In gas phase, different considerations hold. In fact, toluene molecules are way smaller and have easier mobility compared with RhB ones. In this way, nanotubes depth is exploited to a larger extent: it is not surprising then to see much higher photoactivity for O-NT, where tubes are longer, smoother, less obstructed and present less recombination centers compared with A-NT. On the other hand, samples prepared by ASD show negligible activity in this case, since they are strongly penalized by the extremely low surface area.

Such observations underline the utility of having a set of photocatalysts with different morphological and structural characteristics available, since different reactants may find easier decomposition over given substrates but not on others, or differences could be attenuated depending on the reactant nature and reaction paths. This could allow the use of more easy-to-produce ASD photocatalysts in light duty occurrence, such as RhB degradation, while more active photocatalysts, such as organic nanotubes, would be required in more demanding conditions such as gas phase pollutants degradation.

## 5. Conclusions

We presented the production and characterization of nanostructured titanium dioxide photocatalysts, obtained by means of different anodic oxidation techniques. Nanotubes prepared in an organic electrolyte show good photoactivity, in both gas and liquid phase. 

The operating parameters affect the morphology and surface area of the different TiO_2_ nanostructures, and thus their efficiency in pollutants degradation. The effect of the preparation electrolyte on photocatalytic activity is evident: in particular, films with nanotubular morphology present much higher efficiency if produced in organic electrolytes than in aqueous ones, especially in gas phase, where the pollutant can better diffuse through the longer length of the organic nanotubes. The morphology obtained by anodizing at high voltage in absence of fluorides presents a comparable efficiency with aqueous nanotubes when used in liquid phase, and no activity when used in gas phase, in spite of a superior crystallinity: this suggests that surface area is the ruling parameter in gas phase photocatalysis, while in liquid phase, where larger molecules are generally involved—e.g., organic dyes—only a part of the pores length can be exploited, therefore flattening the differences in photocatalytic activities among the three oxides.

## Figures and Tables

**Table 1:** Summary of oxides characteristics as emerges from SEM images.

Sample	Electrolyte	Anodizing Time (min)	Avg Tube Wall Thickness (nm)	Avg Tube Length/Oxide Thickness (μm)
ASD	H_2_SO_4_	2	Does not apply	0.7
A-NT	NaF + Na_2_SO_4_	360	15	1.5
O-NT	NH_4_F + EG	30	30–40	3

**Table 2:** Photocatalytic activity in gas and liquid phase for samples produced in different conditions: light intensity for toluene degradation 160 μW cm^−2^, for RhB degradation 3000 μW cm^−2^.

Activity Indicator	Reference	ASD	A-NT	O-NT
$k_{app}$, RhB (m^−2^ s^−1^)	0.002 ± 0.001	0.054 ± 0.005	0.050 ± 0.005	0.090 ± 0.010
*r_a_*, toluene (nmol m^−2^ s^−1^)	0.00 ± 0.06	0.03 ± 0.06 ^1^	0.14 ± 0.06	1.37 ± 0.11

^1^ This measure was carried out at higher UV-A irradiance (525 µW cm^−2^) in order to confirm the absence of any detectable activity.

## References

[1] Ibhadon A., Fitzpatrick P. (2013). Heterogeneous photocatalysis: Recent advances and applications. Catalysts.

[2] Lazar M., Varghese S., Nair S. (2012). Photocatalytic water treatment by titanium dioxide: Recent updates. Catalysts.

[3] Tompkins D., Lawnicki B., Zeltner W., Anderson M. (2005). Evaluation of photocatalysis for gas-phase air cleaning—Part 1: Process, Technical, and Sizing Considerations. ASHRAE.

[4] Hsien H.-Y., Chang C.-F., Chen Y.-H., Cheng S. (2001). Photodegradation of aromatic pollutants in water over TiO_2_ supported on molecular sieves. Appl. Catal. B Environ..

[5] Einaga H., Mochiduki K., Teraok Y. (2013). Photocatalytic oxidation processes for toluene oxidation over TiO_2_ catalysts. Catalysts.

[6] Piera E., Ayllón J., Doménech X., Peral J. (2002). TiO_2_ deactivation during gas-phase photocatalytic oxidation of ethanol. Catal. Today.

[7] Lisebigler A., Lu G., Yates J. (1995). Photocatalysis on TiO_2_ surfaces: Principles, mechanisms and selected results. Chem. Rev..

[8] Gunti S., Kumar A., Ram M.K. (2018). Nanostructured photocatalysis in the visible spectrum for the decontamination of air and water. Int. Mater. Rev..

[9] Khalid N.R., Majid A., Tahir M.B., Niaz N.A., Khalid S. (2018). Carbonaceous-TiO_2_ nanomaterials for photocatalytic degradation of pollutants: A review. Ceram. Int..

[10] Han E., Vijayarangamuthu K., Youn J.-S., Park Y.-K., Jung S.-C., Jeon K.-J. (2018). Degussa P25 TiO_2_ modified with H_2_O_2_ under microwave treatment to enhance photocatalytic properties. Catal. Today.

[11] Melcher J., Feroz S., Bahnemann D. (2018). Comparing photocatalytic activities of commercially available iron-doped and iron-undoped aeroxide TiO_2_ P25 powders. J. Mater. Sci..

[12] Souza R.P., Freitas T.K.F.S., Domingues F.S., Pezoti O., Ambrosio E., Ferrari-Lima A.M., Garcia J.C. (2016). Photocatalytic activity of TiO_2_, ZnO and Nb_2_O_5_ applied to degradation of textile wastewater. J. Photochem. Photobiol. A Chem..

[13] Alvarez-Corena J.R., Bergendahl J.A., Hart F.L. (2016). Advanced oxidation of five contaminants in water by UV/TiO_2_: Reaction kinetics and byproducts identification. J. Environ. Manag..

[14] Qin X., Jing L., Tian G., Qu Y., Feng Y. (2009). Enhanced photocatalytic activity for degrading Rhodamine B solution of commercial Degussa P25 TiO_2_ and its mechanisms. J. Hazard. Mater..

[15] Ahmed S., Rasul M., Martens W., Brown R., Hashib M. (2011). Advances in heterogeneous photocatalytic degradation of phenols and dyes in wastewater: A review. Water Air Soil Pollut..

[16] Paramasivam I., Jha H., Liu N., Schmuki P. (2012). A Review of photocatalysis using Self-organized TiO_2_ Nanotubes and Other Ordered Oxide Nanostructures. Small.

[17] Grzechulska-Damszel J., Mozia S., Morawski A. (2010). Integration of photocatalysis with membrane processes for purification of water contaminated with organic dyes. Catal. Today.

[18] Sarantopoulos C., Puzenat E., Guillard C., Herrmann J.-M., Gleizes A., Maury F. (2009). Microfibrous TiO_2_ supported photocatalysts prepared by metal-organic chemical vapor infiltration for indoor air and waste water purification. Appl. Catal. B Environ..

[19] Kathaee A., Kasiri M. (2010). Photocatalytic degradation of organic dyes in the presence of nanostructured titanium dioxide: Influence of the chemical structure of dyes. J. Mol. Catal. A Chem..

[20] Bettini L.G., Diamanti M.V., Sansotera M., Pedeferri M.P., Navarrini W., Milani P. (2016). Immobilized TiO_2_ nanoparticles produced by flame spray for photocatalytic water remediation. J. Nanoparticle Res..

[21] Persico F., Sansotera M., Bianchi C.L., Cavallotti C., Navarrini W. (2015). Photocatalytic activity of TiO_2_-embedded fluorinated transparent coating for oxidation of hydrosoluble pollutants in turbid suspensions. Appl. Catal. B Environ..

[22] Strini A., Sanson A., Mercadelli E., Sangiorgi A., Schiavi L. (2013). Low irradiance photocatalytic degradation of toluene in air by screen-printed titanium dioxide layers. Thin Solid Films.

[23] Gnatyuk Y., Smirnova N., Korduban O., Eremenko A. (2010). Effect of zirconium incorporation on the stabilization of TiO_2_ mesoporous structure. Surf. Interface Anal..

[24] Strini A., Sanson A., Mercadelli E., Bendoni R., Marelli M., Dal Santo V., Schiavi L. (2015). In-situ anatase phase stabilization of titania photocatalyst by sintering in presence of Zr^4+^ organic salts. Appl. Surf. Sci..

[25] Diamanti M.V., Ormellese M., Pedeferri M. (2015). Application-wise nanostructuring of anodic films on titanium: A review. J. Exp. Nanosci..

[26] Macak J., Zlamal M., Krysa J., Schmuki P. (2007). Self-organized TiO_2_ nanotube layer as highly efficient photocatalysis. Small.

[27] Riboni F., Nguyen N.T., So S., Schmuki P. (2016). Aligned metal oxide nanotube arrays: Key-aspects of anodic TiO_2_ nanotube formation and properties. Nanoscale Horiz..

[28] Lee K., Mazare A., Schmuki P. (2014). One-dimensional titanium dioxide nanomaterials: Nanotubes. Chem. Rev..

[29] Qin L., Chen Q., Lan R., Jiang R., Quan X., Xu B., Zhang F., Jia Y. (2015). Effect of anodization parameters on morphology and photocatalysis properties of TiO_2_ Nanotube Arrays. J. Mater. Sci. Technol..

[30] Xie Z., Blackwood D. (2010). Effects of anodization parameters on the formation of titania nanotubes in ethylene glycol. Electrochim. Acta.

[31] Raja K., Gandhi T., Misra M. (2007). Effect of water content of ethylene glycol as electrolyte for synthesis of ordered titania nanotubes. Electrochem. Commun..

[32] Haring A., Morris A., Hu M. (2012). Controlling morphological parameters of anodized titania nanotubes for optimized solar energy applications. Mater.

[33] Mena E., de Vidales M.J., Mesones S., Marugán J. (2018). Influence of anodization mode on the morphology and photocatalytic activity of TiO_2_-NTs array large size electrodes. Catal. Today.

[34] Ye Y., Feng Y., Bruning H., Yntema D., Rijnaarts H.H.M. (2018). Photocatalytic degradation of metoprolol by TiO_2_ nanotube arrays and UV-LED: Effects of catalyst properties, operational parameters, commonly present water constituents, and photo-induced reactive species. Appl. Catal. B Environ..

[35] Diamanti M.V., Ormellese M., Pedeferri M. (2010). Alternating current anodizing of titanium in halogen acids combined with anodic spark depositions: Morphological and structural variations. Corros. Sci..

[36] Zhuiykov S. (2013). Nanostructured Semiconductor Oxides for the Next Generation of Electronics and Functional Devices. Properties and Applications, 1st ed.

[37] Xiao P., Zhang Y., Garcia B.S.S., Liu D.C.G. (2008). Nanostructured electrode with titania nanotubes arrays: Fabrication, electrochemical properties, and applications for biosensing. J. Nanosci. Nanotechnol..

[38] Diamanti M.V., Ormellese M., Marin E., Lanzutti A., Mele A., Pedeferri M. (2011). Anodic titanium oxide as immobilized photocatalyst in UV or visible light devices. J. Hazard. Mater..

[39] Wu J.-M., Zhang T.-W. (2004). Photodegradation of rhodamine B in water assisted by titania films prepared through a novel procedure. J. Photochem. Photobiol. A Chem..

[40] Strini A., Schiavi L. (2011). Low irradiance toluene degradation activity of a cementitious photocatalytic material measured at constant pollutant concentration by a successive approximation method. Appl. Catal. B Environ..

[41] Park Y.-J., Shin K.-H., Song H.-J. (2007). Effects of anodizing conditions on bong strength of anodically oxidized film on titanium substrate. Appl. Surf. Sci..

[42] Macak J., Sirotna K., Schmuki P. (2005). Self-organized porous titanium oxide prepared in Na_2_SO_4_/NaF electrolytes. Electrochim. Acta.

[43] Li Bassi A., Cattaneo D., Russo V., Bottani C.E., Barborini E., Mazza T., Piseri P., Milani P., Ernst F.O., Wegner K. (2005). Raman Spectroscopy Characterization of titania nanoparticles produced by flame pyrolysis: The influence of size and stoichiometry. J. Appl. Phys..

[44] Ohno T., Sarukawa K., Tokieda K., Matsumura M. (2001). Morphology of a TiO_2_ photocatalyst (Degussa, P-25) consisting of anatase and rutile crystalline phases. J. Catal..

[45] Siah W.R., Lintang H.O., Shamsuddin M., Yuliati L. (2016). High photocatalytic activity of mixed anatase-rutile phases on commercial TiO_2_ nanoparticles. IOP Conf. Ser. Mater. Sci. Eng..

[46] Hurum D.C., Agrios A.G., Gray K.A., Rajh T., Thurnauer M.C. (2003). Explaining the enhanced photocatalytic activity of Degussa P25 mixed-phase TiO_2_ using EPR. J. Phys. Chem. B.

[47] Diamanti M.V., Pedeferri M. (2007). Effect of anodic oxidation parameters on the titanium oxides formation. Corros. Sci..

[48] Bakardjieva S., Šubrt J., Štengl V., Dianez M.J., Sayagues M.J. (2005). Photoactivity of anatase-rutile TiO_2_ nanocrystalline mixtures obtained by heat treatment of homogeneously precipitated anatase. Appl. Catal. B Environ..

[49] Nečas D., Klapetek P. (2012). Gwyddion: An open-source software for SPM data analysis. Cent. Eur. J. Phys..

[50] Lynch R.P., Ghicov A., Schmuki P. (2010). A photo-electrochemical investigation of self-organized TiO_2_ nanotubes. J. Electrochem. Soc..

